# Proton Pump Inhibitor Use May Increase the Risk of Diverticulitis but Not It’s Severity among Patients with Colonic Diverticulosis: A Multicenter Study

**DOI:** 10.3390/jcm9092966

**Published:** 2020-09-14

**Authors:** Wisam Sbeit, Tawfik Khoury, Anas Kadah, Waseem Asadi, Amir Shahin, Ahmad Lubany, Mohammed Safadi, Haya Haddad, Ruba Abu Ahmad, Sami Abu El Hija, Rand Abboud, Mahmud Mahamid, Rinaldo Pellicano, Amir Mari

**Affiliations:** 1 Department of Gastroenterology, Galilee Medical Center, Nahariya, Israel, Faculty of Medicine in the Galilee, Bar-Ilan University, Safed 13100, Israel; wisams@gmc.gov.il (W.S.); tawfikkhoury1@hotmail.com (T.K.); anask@gmc.gov.il (A.K.); wassim_assadi@yahoo.com (W.A.); amir.schahin@gmail.com (A.S.); 2 Surgery Department, The Nazareth Hospital, EMMS, Nazareth 1613101, Israel; ahmadlubany@gmail.com (A.L.); dr.m.safadi99@gmail.com (M.S.); haya_haddad@hotmail.com (H.H.); Rubaabuahmad@gmail.com (R.A.A.); sami.813@hotmail.com (S.A.E.H.); rand.abboud@gmail.com (R.A.); 3 Faculty of Medicine, Hebrew University of Jerusalem, Department of Gastroenterology and Liver Diseases, Shaare Zedek Medical Center, Jerusalem 9103401, Israel; mahmudmahamid@yahoo.com; 4 Gastroenterology Unit, Molinette Hospital, 10125 Turin, Italy; rinaldo_pellican@hotmail.com; 5 Gastroenterology and Endoscopy United, The Nazareth Hospital, EMMS, Faculty of medicine, Bar-Ilan University, Nazareth 1613101, Israel

**Keywords:** PPI, diverticulosis, diverticulitis, risk factors

## Abstract

Colonic diverticular disease, especially diverticulitis constitutes a major cause of hospitalization and an economic burden in developed countries. Proton pump inhibitors (PPIs) are among the commonest drugs used to treat several diseases affecting the upper gastrointestinal tract. A few studies have reported that the use of Proton Pump Inhibitors PPIs caused dysbiosis. In this study, we searched for a relationship between PPI use and the onset and severity of diverticulitis in patients with colonic diverticulosis. In a retrospective study, patients who were hospitalized for documented diverticulitis were enrolled as cases and compared with a control group of patients with uncomplicated diverticulosis. Overall, 613 patients who had a diagnosis of diverticulosis were included in the study, 217 of whom had diverticulitis. After multivariate analysis, the non-modifiable risk factors associated with diverticulitis included: age (*p* < 0.0001), hypertension (*p* < 0.0001), chronic renal failure (*p* = 0.007), diabetes mellitus (*p* < 0.0001), and left colon location (*p* = 0.02). However, among the modifiable factors, only PPI use (*p* < 0.0001) showed a significant association. Advanced disease severity (according to Hinchey classification of diverticulitis stages II–IV) was associated with aspirin use (*p* = 0.0004) and pan-colonic location (*p* = 0.02). PPI use was the only modifiable factor significantly associated with diverticulitis, but not with its severity, among patients with diverticulosis. This observation should be confirmed in future multicenter prospective studies.

## 1. Introduction

Colonic diverticular disease, especially when complicated by diverticulitis and bleeding, constitutes a major cause of hospitalization and an economic burden in developed countries [1,2]. Complications, such as diverticulitis, bleeding, and perforation occur in about 10–20% of patients with colonic diverticulosis [3]. The incidence of diverticulitis has been increasing, as reported by a nationwide study of hospitalizations performed in the United States that revealed a 26% increase in admissions from 1998 to 2005 [4]. Well-known risk factors for diverticulitis include high intake of red meat or fat, low fiber intake, high body mass index (BMI), lack of physical activity, smoking habits [5], and nonsteroidal anti-inflammatory drug (NSAID) use [6]. The main pathogenetic mechanism seems to involve inspissated fecal material that leads to mucus secretion and eventual bacterial overgrowth within the diverticulum inducing inflammation, focal necrosis, and micro- or macroperforation [7]. Due to their high efficacy and low toxicity, proton pump inhibitors (PPIs) are the most common drug used to treat gastroesophageal reflux disease, peptic ulcer disease (PUD), and to prevent NSAID and aspirin associated peptic ulcer disease [8]. Their use, especially long-term use, has increased significantly [9]. Alongside the gastric H^+^/K^+^-ATPase (proton pump) blocked by PPIs, several bacteria within the gastrointestinal tract produce acid and contain ATPase enzymes. Hence, it is believed that PPIs can inadvertently affect these bacteria directly by targeting their proton pumps and indirectly by changing the pH with an effect on the gut flora [10]. In fact, several studies have reported that PPI use has led to dysbiosis [9,11,12,13]. The role of gut microbiota in host resistance against colonization by exogenous enteric microbes and overgrowth of indigenous commensals is well known [14]. Accordingly, PPI-induced dysbiosis can increase the risk of bacterial enteric infections and translocation [9,15].

In this multicenter retrospective study, we aimed to study whether or not PPI use was associated with increased risk of diverticulitis development among patients with diverticulosis, as well as to assess whether PPI use was related to diverticulitis severity.

## 2. Materials and Methods

This study was performed using the databases of three Israeli academic medical centers (Galilee Medical Center, EMMS Nazareth Hospital, and Sharee Zedek Medical Center), from January 2010 to December 2019. The group consisted of 613 patients, and included patients with diverticulosis who lacked signs of inflammation as confirmed in computed tomography (CT) scans that had been performed electively for various causes and diverticulosis and that had been documented in the final report diagnosis. A second search was performed among these patients to detect patients who had been hospitalized at a later time (more than 6 months from the first elective CT), with the clinical diagnosis of diverticulitis (based on history, physical examination, and inflammatory markers) that had been confirmed by computed tomography (CT) scan, during the study period when they were eligible for enrollment in the study. The group of patients with uncomplicated diverticulosis served as the control.

PPI use was defined as taking PPIs for more than one month during an interval of 6 months prior to diverticulitis.

The extracted data included demographic variables (age and gender), smoking and alcohol drinking habits, as well as PPI, NSAID, aspirin, and statin use. Exclusion criteria resulted in excluding the following patients: 6 patients with a concomitant diagnosis of colorectal cancer and 2 patients who presented with colonic perforation and underwent surgery. The study protocol conformed to the ethical guidelines of the 1975 Declaration of Helsinki [16], and was approved by each institution’s human research committee. Written informed consent was waived by the local ethical committee due to the retrospective non-interventional nature of the study.

### 2.1. Study Aims

The primary aim of the study was to elucidate whether or not PPI use increased the risk of developing an episode of acute diverticulitis among patients with diverticulosis. The secondary aim was to identify predictors of increased diverticulitis severity, as assessed by the Hinchey classification on the basis of CT scan results which were reported as follows: stage 0, mild clinical diverticulitis with mild bowel wall inflammation; stage I, localized abscess (para-colonic); stage II, pelvic abscess; stage III, purulent peritonitis; and stage IV, fecal peritonitis [17]. Patients who presented with Hinchey grade 0 and I were considered to have mild disease, while those with Hinchey grades II, III, and IV (generalized fecal peritonitis) were considered to have moderate to severe complicated diverticulitis.

### 2.2. Statistical Analysis

Chi-square and Fisher’s exact tests were used to analyze the association between two categorical variables (presented as frequencies and percentages), while either the two-sample t-test or the Mann–Whitney U test was used to compare continuous variables (reported as mean ± standard deviation (SD)). To measure the association between PPI exposure and the risk and severity of diverticulitis, a univariate model analysis was performed and variables with statistically significant *p* values (<0.05) by univariate analysis were entered into the multiple logistic regression analysis which reported odds ratios (OR) and confidence intervals (CI). Statistical analyses were carried out with commercial software, Statistical Package for Social Science (SPSS version 24.0, IBM, Chicago, IL, USA).

## 3. Results

### 3.1. Demographics, Clinical, and Laboratory Characteristics

Overall, 613 patients with a confirmed diagnosis of diverticulosis were included in the study. Among them, 217 patients had at least one diverticulitis episode (group A), as compared with 396 patients who did not develop diverticulitis (group B). The mean age was 60.7 ± 14.7 and 70.4 ± 12.3 years in groups A and B, respectively. In group A, 66.8% were males as compared with 59.6% in group B. Notably, among the patients in group A, 120 (55.3%), 13 (6%), 81 (37.3%), and 85 (39.2%) of them were on chronic treatment (more than 1 month) with PPIs, NSAIDs, statins, and aspirin, respectively, as compared with 111 (28%), 23 (5.8%), 186 (47%), and 178 (45%) patients in group B. In all groups, the most common location of diverticulosis was the sigmoid colon (53% vs. 58.8% in groups A and B, respectively). Table 1 shows the demographics and baseline characteristics of the study cohort.

### 3.2. Parameters Associated with Diverticulitis in the Univariate and Multivariate Analysis

We identified several parameters associated with the development of diverticulitis (Table 2). The univariate model analysis indicated that age (OR 0.95, 95% CI 0.94–0.96, *p* < 0.0001), hypertension (OR 0.38, 95% CI 0.27–0.54, *p* < 0.0001), chronic renal failure (OR 0.27, 95% CI 0.11–0.68, *p* = 0.005), heart failure (OR 0.28, 95% CI 0.11–0.71, *p* = 0.007), diabetes mellitus (OR 2.92, 95% CI 2.06–4.13, *p* < 0.0001), obesity (OR 2.55, 95% CI 1.81–3.60, *p* < 0.0001), left colon location (OR 0.61, 95% CI 0.41–0.92, *p* = 0.016), statin use (OR 0.67, 95% CI 0.48–0.95, *p* = 0.02), and PPI use (OR 2.77, 95% CI 1.45–5.32, *p* = 0.002) were statistically significant. Conversely, there was no effect of other parameters including smoking (*p* = 0.12), NSAID use (*p* = 0.89), and aspirin use (*p* = 0.17). In the multivariate logistic regression analysis, age (OR 0.94, 95% CI 0.92–0.95, *p* < 0.0001), hypertension (OR 0.25, 95% CI 0.15–0.44, *p* < 0.0001), chronic renal failure (OR 0.23, 95% CI 0.08–0.68, *p* = 0.007), diabetes mellitus (OR 5.73, 95% CI 3.11–10.52, *p* < 0.0001), and left colon location (OR 0.58, 95% CI 0.35–0.94, *p* = 0.02) remained significantly correlated with diverticulitis, whereas among the modifiable risk factors, only PPI use (OR 3.94, 95% CI 2.26–6.86, *p* < 0.0001) was significantly associated with diverticulitis (Table 3).

### 3.3. Parameters Associated With Diverticulitis Severity

Overall, 217 patients had an episode of diverticulitis, however, since two patients had missing data, the final analysis was performed on 215 patients. Among them, 59 patients (27.2%) had moderate to severe diverticulitis defined by Hinchey stage II–IV (group C) as compared with 156 patients (72.8%) who had mild diverticulitis (stage 0–I) (group D). The mean ages for groups C and D were 65.8 ± 11.8 and 58.8 ± 15.3, respectively. Male gender was similar between the groups (67.8% vs. 66.7%, for groups C and D, respectively). Table 4 shows the baseline characteristics of the cohort with diverticulitis according to severity.

### 3.4. Parameters Associated With Diverticulitis Severity in the Univariate and Multivariate Analysis

The univariate analysis indicated that age (OR 1.04, 95% CI 1.01–1.06, *p* = 0.002), hypertension (OR 3.58, 95% CI 1.89–6.77, *p* < 0.0001), diabetes mellitus (OR 4.01, 95% CI 2.07–7.78, *p* < 0.0001), obesity (OR 4.12, 95% CI 2.12–7.99, *p* < 0.0001), aspirin use (OR 4.26, 95% CI 2.26–8.01, *p* < 0.0001), NSAID use (OR 3.31, 95% CI 1.06–10.28, *p* = 0.04), PPI use (OR 2.77, 95% CI 1.45–5.32, *p* = 0.002), and pan-colonic diverticulosis (OR 3.78. 95% CI 1.88–7.61, *p* = 0.0002) were associated with more severe diverticulitis (Hinchey stage II–IV) (Table 5). After multivariate analysis, only aspirin use (OR 3.4, 95% CI 1.73–6.63, *p* = 0.0004) and pan-colonic location (OR 2.41, 95% CI 1.13–5.13, *p* = 0.02) remained significantly associated with severe diverticulitis (Hinchey stage II–IV) (Table 5), whereas there was no effect of NSAID use (*p* = 0.2), PPI use (*p* = 0.11), age (*p* = 0.7), hypertension (*p* = 0.1), diabetes mellitus (*p* = 0.25), and obesity (*p* = 0.1).

## 4. Discussion

Colonic diverticulosis is becoming one the most common diseases worldwide [18,19]. Although most patients have a silent asymptomatic course throughout their life, an increasing prevalence of diverticulitis with increasing morbidity has been encountered, posing a significant burden on health service systems [20], with approximately 20% of patients needing surgical intervention for diverticulitis at first presentation [21,22]. The increased prevalence of diverticulitis cannot be attributed solely to the known risk factors including Western diet (with high red meat and fat intake and low fiber intake), obesity, lack of physical activity, smoking, and NSAID consumption. Therefore, other modifiable risk factors, including medications, could be involved in the development of diverticulitis. Due to the widespread availability and the increased use of PPIs, and the increasing prevalence of diverticulitis, and because both are associated with enteric bacterial overgrowth and infections, a possible causal relationship between PPI use and the development of diverticulitis has been hypothesized. Medications which suppress gastric acid production increase the risk of infections by different pathogens [23], due to hypochlorhydria when the gastric pH exceeds 4.0, enabling pathogens to escape the defense mechanism of the acidic stomach which leads to colonization and bacterial overgrowth, in addition to reduced gastrointestinal host defense originating from delayed gastric emptying, increased bacterial translocation, decreased gastric mucus viscosity, changes in normal microbial flora [24], and blocking the bacterial proton pumps [10]. It has been shown that gastric acid inhibitors increase the risk of community-acquired pneumonia and acute gastroenteritis in children, as a result of changes in the normal gut flora and leukocyte dysfunction [15]. An association between dosage and treatment duration of PPI therapy and increased risk of gastrointestinal infections has been shown [25].

In our study, we showed a possible association between PPI use and the risk of diverticulitis that remained significant in multivariate analysis (*p* > 0.001, OR 3.94, 95% CI 2.26–6.86). This observation was not documented in the English literature hitherto. Notably, major points should be discussed regarding this possible association since demographics such as age and background illnesses could have contributed to this observed association. It is well known that age is a risk factor for diverticulosis and its complications including diverticulitis [6]. The patients in the diverticulitis group were older by about 10 years as compared with the diverticulosis group, a fact that could raise an option of possible confounding. Importantly, the association of PPI use with acute diverticulitis was observed in the multivariate analysis as well. Moreover, the patients in the different groups still belonged to approximately the same age group (60–70), therefore generally speaking an elderly patient group. The only report that has analyzed this matter was a nested case-control study which enrolling 690 patients based on the National Health Insurance Research Database in Taiwan, in which the authors showed that the use of PPIs did not increase the risk of diverticulitis. However, among the limitations of this study, the authors stated that the results could not be applied to Western societies as colonic diverticular disease is not as common in Asian as compared with Western societies [24]. A finding of our multicenter study is that PPI use did not affect the severity of diverticulitis. However, a recent study by Tursi et al. showed that PPI use was directly related to the severity of diverticular disease [26].

Conversely, in our study, some known risk factors for diverticulitis including smoking, as well as NSAID and aspirin use did not affect the rate of diverticulitis. In a study on 47,210 U.S. men included in the Health Professionals Follow-up Study cohort, Strate et al. reported that regular use of aspirin or NSAIDs increased the risk of diverticulitis [6]. Two studies by Laine et al. reported diverticulitis among the commonest serious lower gastrointestinal side effects of NSAIDs [27,28]. These difference with respect to our findings could be explained considering the small number of NSAID users (6%) included in our cohort. However, patients could have used over-the-counter NSAIDs, a fact that could result in data bias.

Furthermore, we found several other known non-modifiable risk factors for the development of diverticulitis in multivariate analysis including advanced age, hypertension, chronic renal failure, diabetes mellitus, and left colon location. Our results were in agreement with the evidence published throughout the literature showing that age [29], hypertension [30], and chronic kidney disease [31] were significantly associated with increased prevalence of diverticulitis. Although there was no data for the association of diabetes mellitus with diverticulitis, diabetes mellitus was associated with advanced disease severity as assessed by Hinchey and Ambrosetti scores [32]. Some possible explanations of this observation are the probable coexistence of DM with metabolic syndrome that can induce the development of diverticulitis through the release of cytokines that promote inflammation in the subcutaneous abdominal fat, as well as alterations in gut microbiota that can play a key role in inducing diverticulitis [33].

Our finding that left colon location had a significant association with diverticulitis is similar to findings reported by previous studies performed in Western countries [34]. Although previous evidence showed that obesity, smoking, and statin use increased the prevalence and severity of diverticular disease and its related complications [35,36], our results did not support the aforementioned findings, suggesting that more studies are warranted to exactly assess this association.

Moreover, although aspirin use did not affect the prevalence of diverticulitis in our study, it was associated with higher diverticulitis severity, as shown by higher Hinchey classification. Potential mechanisms for aspirin and NSAID diverticular complications are supposed to result from direct topical injury to the colon and impaired prostaglandin synthesis which compromise mucosal integrity, increase permeability, and enable the influx of bacteria and toxins [37]. Therefore, more data from laboratory research and prospective studies are needed to elucidate the exact effect of aspirin and NSAIDs on the prevalence and severity of diverticulitis.

The main limitation of our study is its retrospective design that could cause data and information bias. Ideally, the possible causal relationship between PPI and diverticulitis should be assessed in a prospective study design rather than retrospectively. However, a strength of our study included limited potential data heterogeneity, due to the fact that, in all three involved centers, all the physicians followed homogeneous protocols for both data collection and diagnostic algorithm. Furthermore, during the last 20 years, all consultations had been recorded in a computerized data bank, and therefore our data were complete and homogeneous.

Another main strength of this study was the multicenter design and the relatively large number of patients included.

## 5. Conclusions

In conclusion, several modifiable and non-modifiable factors were found to be associated with an increased prevalence of colonic diverticulitis. PPI use seems to be associated with acute diverticulitis but not its severity. Moreover, disease severity was mainly affected by aspirin use and pan-colonic location. Larger prospective studies are warranted in order to confirm our data.

## Figures and Tables

**Table 1:** Demographics and baseline characteristics of study cohort.

Parameters	Patients with Diverticulitis	Patients without Diverticulitis
Number of Patients	217	396
Age (Years), Mean ±SD (Range)	60.7 ± 14.7 (24–88)	70.4 ± 12.3 (18–97)
***Gender n (%)***		
Male	145(66.8)	236(59.6)
Female	72(33.2)	160(40.4)
***Medical History, n (%)***		
Hypertension	103(47.5)	279(70.5)
Diabetes Mellitus	110(50.7)	103(26)
Smoking	58(26.7)	90(22.7)
Ischemic Heart Disease	34(15.7)	81(20.5)
Chronic Renal Failure	5(2.3)	34(8.6)
Heart Failure	5(2.3)	33(8.3)
Obesity (BMI > 30)	109(50.2)	112(28.3)
Proton Pump Inhibitors Use, *n* (%)	120(55.3)	111(28)
Non-Steroidal Anti-Inflammatory Drugs Use, *n* (%)	13(6)	23(5.8)
Statins Use, *n* (%)	81(37.3)	186(47)
Aspirin Use, *n* (%)	8(39.2)	178(45)
***Site of Diverticulosis, n (%)***		
Sigmoid	115(53)	233(58.8)
Left Colon	42(19.3)	112(28.3)
Transverse Colon	0	11(2.8)
Right Colon	20(9.2)	29(7.3)
Pan-Colonic	43(19.8)	81(20.4)

**Table 2:** Univariate analysis of parameters associated with diverticulitis.

Parameter	Odds Ratio	95% Confidence Interval	*p* Value
Age (Years)	0.95	0.94–0.96	*<0.0001*
Gender (Male Vs. Female)	1.36	0.96–1.93	0.08
Hypertension	0.38	0.27–0.54	*<0.0001*
Chronic Renal Failure	0.27	0.11–0.68	*0.005*
Heart Failure	0.28	0.11–0.71	*0.007*
Ischemic Heart Disease	0.73	0.47–1.13	0.16
Diabetes Mellitus	2.92	2.06–4.13	*<0.0001*
Obesity	2.55	1.81–3.60	*<0.0001*
Smoking	1.33	0.91–1.95	0.15
Statins Use	0.67	0.48–0.95	*0.02*
Non-Steroidal Anti-Inflammatory Drugs Use	1.05	0.52–2.11	0.89
Aspirin Use	0.79	0.56–1.11	0.17
Proton Pump Inhibitors Use	2.77	1.45–5.32	*0.002*
Sigmoid Location	0.79	0.57–1.10	0.16
Left Colon Location	0.61	0.41–0.92	0.016
Transverse Colon Location	0.08	0.004–1.49	0.09
Right Colon Location	1.29	0.71–2.34	0.4
Pan-Colonic Location	0.97	0.64–1.46	0.87

**Table 3:** Multivariate analysis of parameters associated with diverticulitis.

**Non- Modifiable Risk Factors**			
Parameter	**Odds Ratio**	**95% Confidence Interval**	***p* Value**
Age (Years)	0.94	0.92–0.95	<0.0001
Hypertension	0.25	0.15–0.44	*<0.0001*
Chronic Renal Failure	0.23	0.08–0.68	0.007
Diabetes Mellitus	5.73	3.11–10.52	<0.0001
Left Colon Location	0.58	0.35–0.94	0.02
**Modifiable Risk Factors**			
**Parameter**	**Odds Ratio**	**95% Confidence Interval**	***p* value**
Proton Pump Inhibitors Use	3.94	2.26–6.86	<0.0001

**Table 4:** Demographics and baseline characteristics of the study cohort with diverticulitis.

Parameters	Severe Diverticulitis (Hinchey Stage II-IV)	Mild Diverticulitis (Hinchey Stage 0-I)
Number of Patients	59	156
Age (Years), Mean ±SD (Range)	65.8 ± 11.8(25–88)	58.8 ± 15.3(24–87)
Gender n (%)		
Male	40(67.8)	104(66.7)
Female	19(32.2)	52(33.3)
Medical History, n (%)		
Hypertension	41(69.5)	60(38.5)
Diabetes Mellitus	44(74.6)	65(41.7)
Smoking	18(30.5)	39(25)
Ischemic Heart Disease	13(22)	20(12.8)
Chronic Renal Failure	3(5.1)	2(1.3)
Heart Failure	3(5.1)	2(1.3)
Obesity	44(74.6)	64(41)
Proton Pump Inhibitors Use, n (%)	43(72.9)	76(487)
Non-Steroidal Anti-Inflammatory Drugs Use, n (%)	7(11.9)	6(3.9)
Statins Use, n (%)	27(45.8)	53(34)
Aspirin Use, n (%)	38(64.4)	46(29.5)
Site of Diverticulosis, n (%)		
Sigmoid	25(42.4)	89(57.1)
Left Colon	9(15.3)	31(19.9)
Right Colon	3(5.1)	17(10.9)
Pan-Colonic	22(37.3)	21(13.5)

**Table 5:** Univariate and multivariate analyses of parameters associated with higher diverticulitis severity (Hinchey stage II–IV).

**Univariate Analysis**			
**Parameter**	**Odds Ratio**	**95% Confidence Interval**	***p* Value**
Age (Years)	1.04	1.01–1.06	*0.002*
Gender Male Vs. Female	1.04	0.55–1.97	0.9
Hypertension	3.58	1.89–6.77	*<0.0001*
Chronic Renal Failure	3.83	0.63–23.25	0.14
Heart Failure	3.83	0.63–23.25	0.14
Ischemic Heart Disease	1.93	0.89–4.19	0.09
Diabetes Mellitus	4.01	2.07–7.78	*<0.0001*
Obesity	4.12	2.12–7.99	*<0.0001*
Smoking	1.24	0.64–2.42	0.52
Statins Use	1.64	0.89–3.01	0.11
Non-Steroidal Anti-Inflammatory Drugs Use	3.31	1.06–10.28	*0.04*
Aspirin Use	4.26	2.26–8.01	*<0.0001*
Proton Pump Inhibitors Use	2.77	1.45–5.32	*0.002*
Sigmoid Location	0.56	0.31–1.02	0.058
Left Colon Location	0.75	0.34–1.68	0.48
Right Colon Location	0.49	0.15–1.67	0.25
Pan-Colonic Location	3.78	1.88–7.61	*0.0002*
**Multivariate Logistic Regression Analysis**			
**Parameter**	**Odds Ratio**	**95% Confidence Interval**	***p* value**
Aspirin Use	3.4	1.73–6.63	*0.0004*
Pan-Colonic Location	2.41	1.13–5.13	*0.02*

## Abbreviations

PPIs	Proton Pump Inhibitors
BMI	Body Mass Index
NSAIDs	Nonsteroidal Anti-Inflammatory Drugs
PUD	Peptic Ulcer Disease
CT	Computed Tomography
OR	Odds Ratio
CI	Confidence Interval
DM	Diabetes Miletus
